# Resistance of House Fly, *Musca domestica* L. (Diptera: Muscidae), to Five Insecticides in Zhejiang Province, China: The Situation in 2017

**DOI:** 10.1155/2019/4851914

**Published:** 2019-06-23

**Authors:** Jin-Na Wang, Juan Hou, Yu-Yan Wu, Song Guo, Qin-Mei Liu, Tian-Qi Li, Zhen-Yu Gong

**Affiliations:** Zhejiang Provincial Center for Disease Control and Prevention, 3399 Binsheng Road, Binjiang District, Hangzhou 310051, China

## Abstract

**Objectives:**

High dependency on pesticides could cause selection pressure leading to the development of resistance. This study was conducted to assess the resistance of the house fly, *Musca domestica*, to five insecticides, namely, permethrin, deltamethrin, beta-cypermethrin, propoxur, and dichlorvos, in Zhejiang Province.

**Methods:**

Field strains of house flies were collected from the 12 administrative districts in Zhejiang Province in 2011, 2014, and 2017, respectively. Topical application method was adopted for the bioassays. The probit analysis was used to determine the median lethal doses with the 95% confidence interval, and then the resistance ratio (RR) was calculated. The insecticides resistance in different years and the correlations of the resistance between different insecticides were also analyzed.

**Results:**

The resistance of field strains house flies to insecticides in Zhejiang Province was relatively common, especially for permethrin, deltamethrin, and beta-cypermethrin. The reversion of the resistance to dichlorvos was found, and most of the field strains in Zhejiang Province became sensitive to dichlorvos in 2017. Propoxur was much easier to cause very high level of resistance; the Hangzhou strain had the highest RR value more than 1000 in 2014, and five field strains had the RR value more than 100 in 2017. Compared to 2011 and 2014, the resistance of the house flies to propoxur and deltamethrin increased significantly in 2017. The resistance of permethrin, deltamethrin, beta-cypermethrin, and propoxur was significantly correlated with each other, and the resistance of dichlorvos was significantly correlated with beta-cypermethrin.

**Conclusions:**

Our results suggested that resistance was existed in permethrin, deltamethrin, beta-cypermethrin, and propoxur in the house flies of Zhejiang Province, while the resistance reversion to dichlorvos was found.

## 1. Introduction

The house fly, *Musca domestica* L. (Diptera: Muscidae), is one of the major public health pests responsible for the transmission of more than 100 pathogens of humans, poultry, and livestock [1–3]. Through contact with carcasses, excreta, garbage, and other septic matter, the house fly has close association with pathogens and plays a role in the mechanical transmission of pathogens (e.g., bacterial, protozoan, helminthic, and viral infections) to humans as well as domesticated animals [4]. Furthermore, diarrheal diseases and avian influenza transmitted by the house fly can cause human death, while high density of flies can reduce the aesthetic value of livestock products and usually brings economic losses [5, 6].

Being fast acting, cheap and convenient, insecticides are often the preferred choice for controlling pests, including house flies. Globally, chemical insecticides such as pyrethroids, organophosphates, and carbamates have been used to control house flies [7]. Particularly in Zhejiang Province, some of the products such as dichlorvos, permethrin, deltamethrin, beta-cypermethrin, and propoxur have also been widely used. Although insecticide application is effective in reducing house flies density, high dependency on pesticides can cause selective pressure and further can lead to the development of resistance [4, 8]. Due to the abuse of insecticides and cross resistance, even the efficiency of a new insecticide applied in pest controlling is limited [1, 9]. Zhejiang is an eastern coastal province of China, dominated by subtropical monsoon climate, which is relatively moderate to the growth and reproduction of house flies. In recent years, avian influenza A (H_7_N_9_) has broken out in Zhejiang Province [10]. Besides, along with infectious diseases including dengue fever, severe fever with thrombocytopenia syndrome et al., [11, 12] and the national patriotic health campaign, a national public health movement in China aiming at the promotion of people's health, the use of a large amount of insecticides in vector control may cause the development of resistance. Therefore, assessment of insecticide resistance risk was important in resistance management strategy application to maintain susceptibility in field strains of the house flies and to sustain the efficacy of these insecticides. The result might have public health implications to guide the use of insecticides and to delay the resistance development [5]. The aims of this study were to assess the resistance levels of the five insecticides of permethrin, deltamethrin, beta-cypermethrin, propoxur, and dichlorvos in house flies, to collect the baseline data for further monitoring and to guide the rational use of the insecticides in Zhejiang Province.

## 2. Materials and Methods

### 2.1. Insect Collection and Feeding

The susceptible strain was originally introduced from the Chinese Center for Disease Control and Prevention (CDC) and reared for more than 30 generations in our laboratory without any insecticide exposure. Field strain house flies were collected from 12 administrative districts in Zhejiang Province, including Hangzhou, Ningbo, Wenzhou, Jiaxing, Shaoxing, Jinhua, Quzhou, Zhoushan, Lishui, Huzhou, Taizhou, and Yiwu. Within each district, 3∼5 sites were selected where the chemical insecticides were frequently used for the management of noisy or health-concerning insects including house flies, and each site more than 100 live house flies were collected by using sweep net. All the flies were maintained in controlled laboratory conditions [13].

### 2.2. Types of Insecticides

Propoxur (98.5%), dichlorvos (95%), permethrin (99%), deltamethrin (96.85%), and beta-cypermethrin (95%) were tested in the toxicological evaluation experiments. The acetone solution (≥99.5%) was applied as a solvent, and the acetone solution alone was applied as a control. The insecticides were provided by the Chinese CDC.

### 2.3. Procedures of Bioassays

The insecticides resistance risk was assessed three times in 2011, 2014 and 2017, respectively. All flies were tested at laboratory conditions with temperature maintained at 25 ± 1°C and relative humidity maintained at 60%∼80%. Topical application method used for the bioassays was from test methods of fIy resistance to insecticides, the bioassay methods for *Musca domestica* [14], which referred to the WHO Pesticide Evaluation Scheme [15]. The F1 generation female adult flies after 3∼5 days of eclosion which had the weight between 18 mg∼20 mg were tested. 30 female flies were tested per dose, and 5∼7 doses was designed for each insecticide. 1 *μ*l of insecticide in acetone solution was applied on thoracic notum of the tested flies, and treated flies were transferred into plastic jars (250 ml) and reared with a cotton dental wick soaked with 20% sugar solution [9, 14]. Each test was replicated three times, and flies treated with acetone alone acted as controls. Mortality counts were made 24 hours after the treatment.

### 2.4. Statistical Analysis

The sensitive baseline method was used to detect the resistance. Probit analysis was used to determine the median lethal doses (LD_50_) with the 95% confidence interval (CI) of the insecticides tested. Kruskal–Wallis H test was used to analyze the resistance to the insecticides in different years. Spearman rank correlation was used to analyze the correlation of the resistance between different insecticides. Among them, resistance ratio (RR) was calculated as follows [1]:

(1) $$\begin{array}{c} \text{RR}=\frac{{\text{LD}}_{50}\text{ of the field strain}}{{\text{LD}}_{50}\text{ of the susceptible reference strain}}. \end{array}$$

The resistant population was defined with the RR value ≥5 and no overlap between the 95% CIs of the tested strain and the susceptible strain [14]. For a high level of resistance, we combined the criterion of Shah et al., [16], defined as very low resistance (RR = 5–10), low resistance (RR = 11–20), moderate resistance (RR = 21–50), high resistance (RR = 51–100), and very high resistance (RR > 100). All the data were analyzed with SPSS16.0 software and a value of <0.05 was considered to be statistically significant.

## 3. Results

### 3.1. General Description

**Figure 1:**
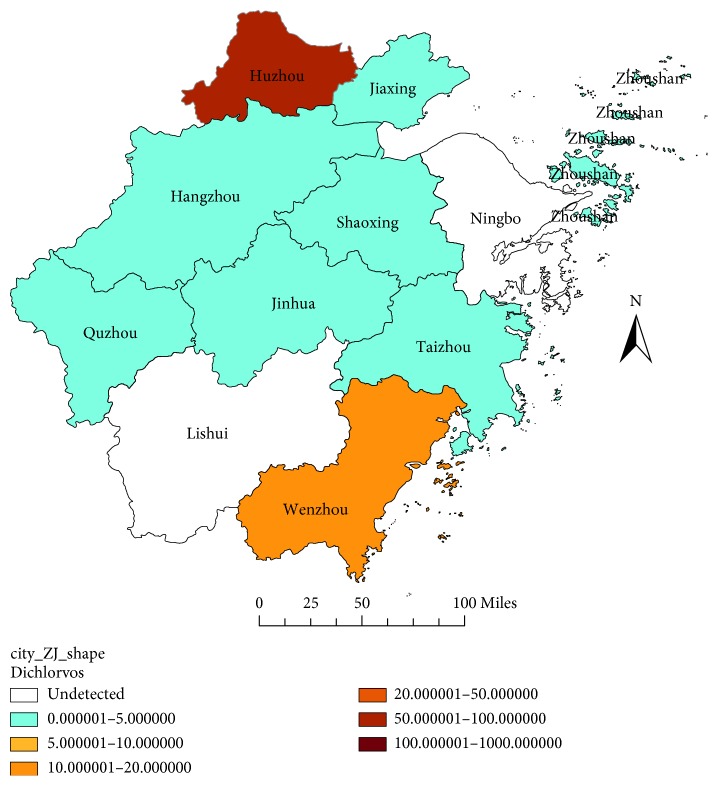
RR value of the house flies to dichlorvos in Zhejiang Province in 2017.

**Figure 2:**
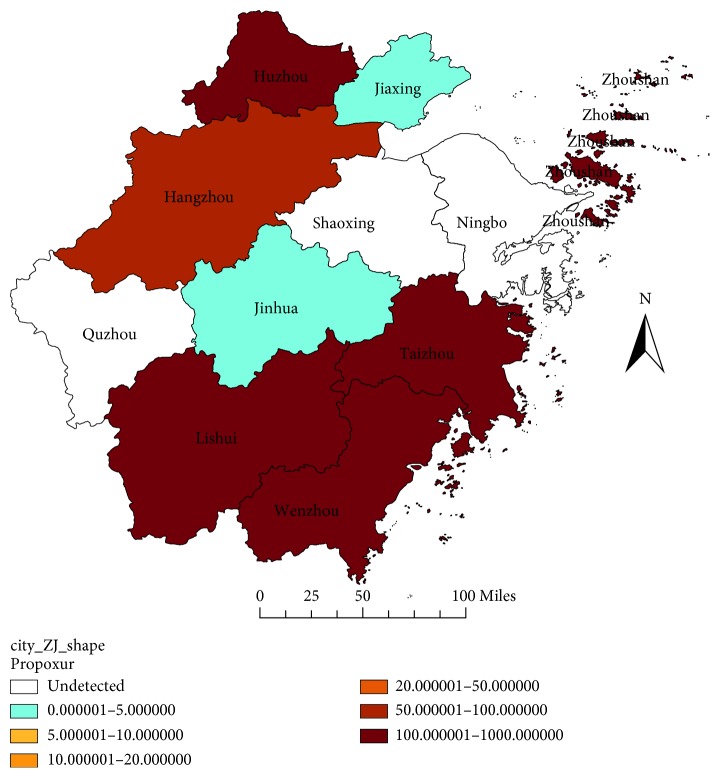
RR value of the house flies to propoxur in Zhejiang Province in 2017.

**Figure 3:**
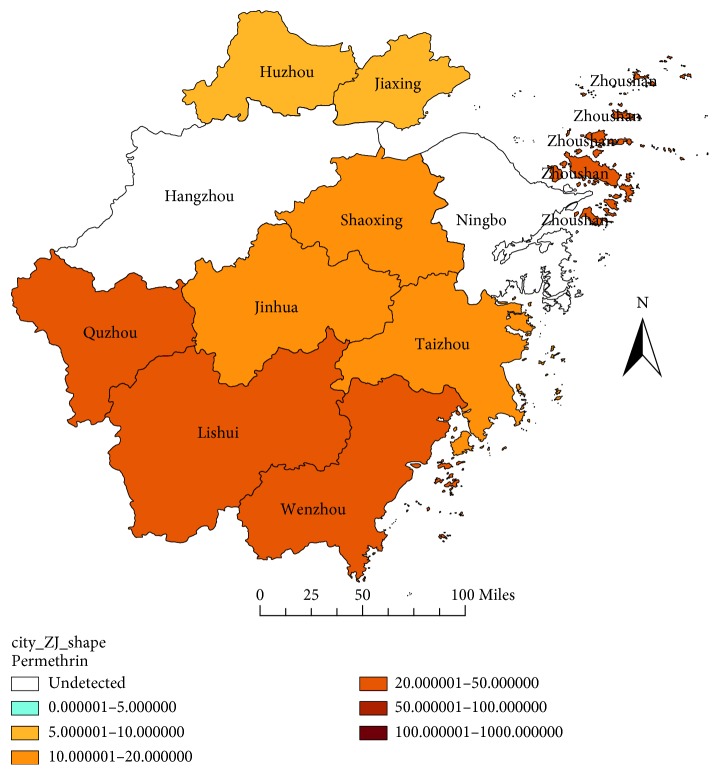
RR value of the house flies to permethrin in Zhejiang Province in 2017.

**Figure 4:**
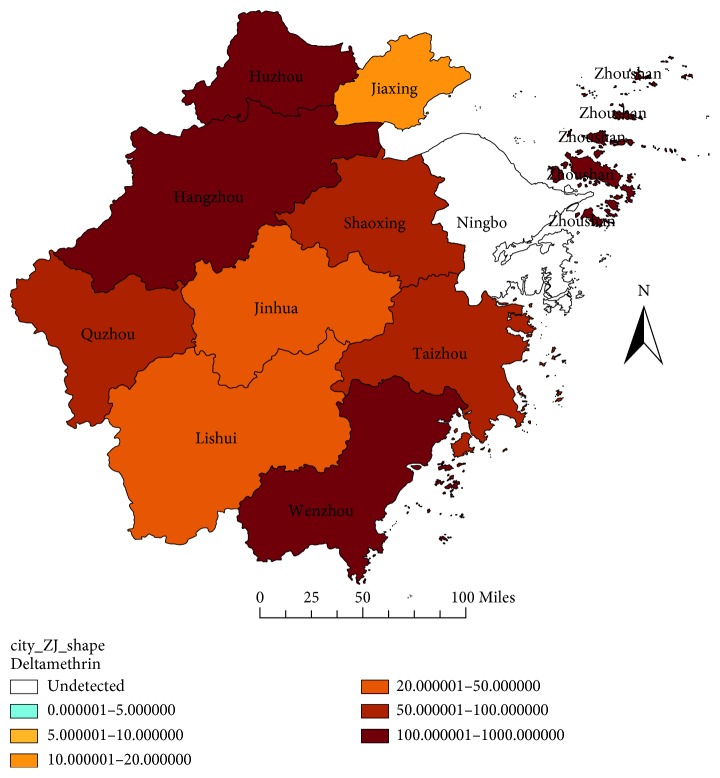
RR value of the house flies to deltamethrin in Zhejiang Province in 2017.

**Figure 5:**
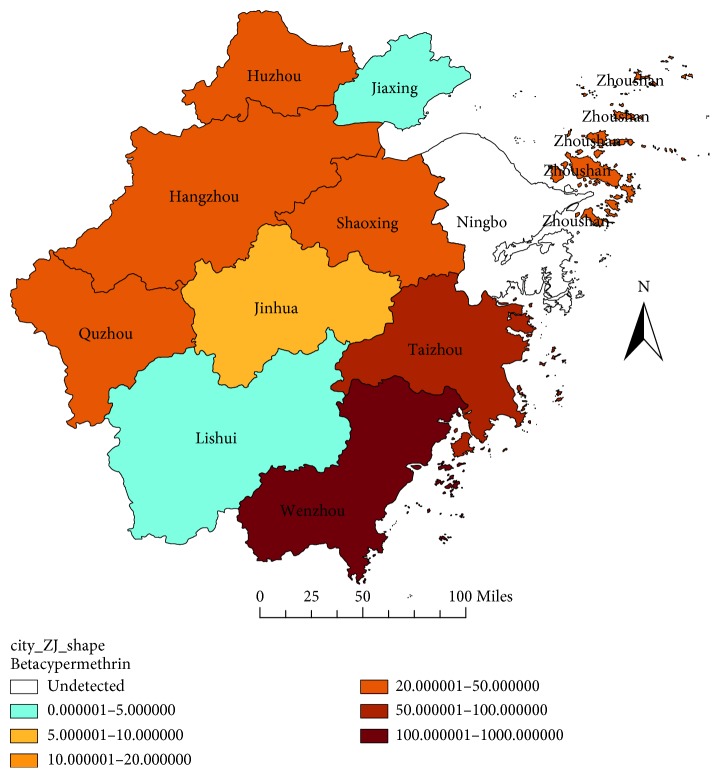
RR value of the house flies to beta-cypermethrin in Zhejiang Province in 2017.

LD_50_ and the RR value of different field strains to the five insecticides in Zhejiang Province are shown in Table 1. The RR value of the 11 prefecture-level cities in 2017 are shown in Figures 1to 5. Yiwu, as a county, is affiliated to Jinhua, and the specific RR value was not shown on the map. Most field strains detected in Zhejiang Province were becoming relatively tolerant with dichlorvos in 2017, apart from the Huzhou and Wenzhou strain (Figure 1). Although the resistance to Ningbo strain was not detected in 2017, tolerance to dichlorvos in the year 2011 and 2014 was seen. The RR value of the Hangzhou field strain to dichlorvos showed a decreasing trend from 15.3316 to 1.3638 in the past three detections (Table 1).

The propoxur was extremely vulnerable to very high resistance. It was noteworthy that LD_50_ of some field strains to the propoxur were too high to be accurately measured. The RR value for the propoxur on Hangzhou strain was more than 1000 in 2014, the RR value of the Lishui strain and Taizhou strain was more than 700 in 2017, the RR value of the Zhoushan strain and Wenzhou strain was more than 300 in 2017, and the RR value of the Huzhou strain was 154.0477 in 2017. Although high levels of resistance were common, there were still some field flies strains sensitive to propoxur in 2017, such as Jiaxing strain, Jinhua strain, etc (Figure 2).

For permethrin, most field strains tested were resistant, apart from the flies strains of Huzhou and Jiaxing in 2014, and the flies strains of Ningbo in 2011 and 2014. The very high level of resistance to permethrin was not found, and the RR value of the field strains to permethrin was below to 50 (Figure 3). For deltamethrin, all flies strains detected were resistant in 2017, and four strains had an RR value more than 100. For example, the Wenzhou strain had the RR value of 375.1429, the Zhoushan strain had the RR value of 163.1429, the Huzhou strain had the RR value of 157.1429, and the Hangzhou strain had the RR value of 111.0000 (Figure 4). For beta-cypermethrin, most strains were resistant in 2017, apart from the sensitive strains of Jiaxing, Lishui, and Yiwu, and the Wenzhou strain had the highest RR value of 364.6444 (Figure 5).

In general, the Ningbo strain had the lowest resistance level in 2011 and 2014, and the resistance was only found in deltamethrin with the RR value 5.8889 in 2014. The Jiaxing strain was still sensitive to certain insecticides such as dichlorvos, propoxur, and beta-cypermethrin in 2017, and the resistance insecticides were permethrin and deltamethrin with the RR value of 8.1296 and 10.5714, respectively. The Huzhou strain and Wenzhou strain had resistance to all the five insecticides in 2017, with very high-level resistance found in these two cities. The RR value of Wenzhou strain to propoxur, deltamethrin, and beta-cypermethrin was more than 300, and the RR value of Huzhou strain to propoxur and deltamethrin was 154.0477 and 157.1429, respectively.

### 3.2. Analysis of the Insecticides Resistance in Different Years

The significant difference was found in the resistance to propoxur and deltamethrin, respectively, in the past three tests (*P* < 0.05) (Table 2). Compared to 2011 and 2014, the resistance of the house flies to propoxur and deltamethrin increased notably in 2017 (*P* < 0.05).

### 3.3. Correlation of the Resistance between Different Insecticides

The correlation of the resistance between different insecticides is shown in Table 3. The RR value of beta-cypermethrin had correlation with dichlorvos, propoxur, permethrin, and deltamethrin, and the *r*_s_ was 0.490, 0.504, 0.467, and 0.734, respectively (*P* < 0.05). Besides, the deltamethrin had correlation with permethrin and propoxur with the *r*_s_ of 0.586 and 0.782, respectively, and permethrin had correlation with propoxur with the *r*_s_ of 0.711 (*P* < 0.05).

## 4. Discussion and Conclusion

With emerging insecticides for the pest control, insecticides resistance management in house flies has become increasingly important. Based on the collected data in 2011, 2014, and 2017, we found that the resistance of the field strains house flies to five insecticides in Zhejiang Province was relatively spread, especially for three of them, namely, permethrin, deltamethrin, and beta-cypermethrin. The propoxur was easier to cause high level of resistance. The reversion of the resistance to dichlorvos was found. House flies' resistance to propoxur and deltamethrin increased notably in 2017. And the cross resistance might exist between certain insecticides.

According to the criterion, we found the resistance to permethrin, deltamethrin, and beta-cypermethrin was common in the field strains. All flies strains tested were resistant to permethrin and deltamethrin in 2017. Compared to 2011 and 2014, the resistance to deltamethrin had a significant rise and came up to a very high level in 2017. To beta-cypermethrin, although some field strains still appeared sensitive, very high-level resistance appeared in 2017. By contrast, the resistance level to permethrin was relatively low with all the RR values below 50 in the last three detections in Zhejiang Province from 2011 to 2017. A study conducted in Turkey showed that pyrethroids resistance changed from spring to fall in relation to usage and application frequencies of these insecticides [17]. The high level of resistance to beta-cypermethrin and deltamethrin in some field strains might be due to the extensive usage of these insecticides. Totally, pyrethroid usage was more common than other insecticides in Zhejiang Province in recent years. The wide application of pyrethroid might contribute to the rise of the house flies resistance.

Very high level of resistance in field populations was also found in propoxur, although some flies strains were still sensitive in Zhejiang Province. The RR value of propoxur in five strains was more than 100 in 2017, in which two strains were more than 700. One strain had the RR value more than 1000 in 2014, indicating the complete uselessness of the propoxur in house flies control. Very high level of resistance to propoxur had been reported before, and the RR value of the resistant strain to propoxur was found greater than 1189.48 compared with the susceptible strain by Chao et al. [18]. In field strains, recessive or dominant expression of the resistance depended on the type and dosage of pesticides used. Research had demonstrated that resistances inherited by a single gene developed more rapidly than those controlled by two or more genes [19], while the house flies resistance to propoxur was inherited as a single, major, autosomal, and incompletely recessive factor [18], which might be the reason for the high level of resistance to propoxur.

Studies have found that house flies resistance to some insecticides was unstable and reversion might be found after several generations, but not to other insecticides [16, 20, 21]. Compared to the susceptible strain, some researchers found that certain insecticide resistance strains had a lower fecundity, hatchability, number of next-generation larvae, and net reproductive rate [16, 20, 22]. The resistance level might be decreased without the selection pressure of insecticides since the resistance alleles could be disadvantageous under natural selection [22]. In China, dichlorvos had been forbidden to use for controlling sanitary pest since 2008. As residual exposure, the reversion of the resistance in the house flies was obviously found, especially in Hangzhou field strain. Most of the field strains in Zhejiang Province became sensitive to dichlorvos in 2017, except the Huzhou and Wenzhou strains. The resistance maintained in this two field strains might be due to the fact that dichlorvos was still slightly used in controlling agriculturally insect pests. Besides, the cross resistance among dichlorvos and other insecticides might be another reason. In general, the resistance reversion to dichlorvos occurred in most of the field strains in Zhejiang Province over the past decade.

Cross resistance among the insecticides has raised concerns in the pesticide application [9, 13]. The correlations among permethrin, deltamethrin, and beta-cypermethrin might indicate the cross resistance between these insecticides. The resistance and cross resistance of house flies between certain pyrethroid insecticides had been reported in previous studies [9, 23]. The major explanation to pyrethroid resistance might be metabolic detoxification, decreased target site sensitivity, and decreased cuticular penetration [2]. The voltage-sensitive sodium channel mutations and increased detoxification mediated by cytochrome P450 monooxygenases overexpression might be the main reason for the resistance [4, 8, 22]. Sun et al. revealed that addition of T929I to the kdr mutation increased resistance to 13 different pyrethroids including permethrin, deltamethrin, and cypermethrin [24], which indicated that these pyrethroids might have cross resistance with each other. Besides, the correlation between propoxur and the pyrethroid insecticides should be noted, and the *r*_s_ were 0.782, 0.711, and 0.504 with deltamethrin, permethrin, and beta-cypermethrin, respectively. The beta-cypermethrin had correlation with dichlorvos with the *r*_s_ 0.490. These correlations might indicate the cross resistance or noncausal association between the insecticides, which need to be confirmed in further study. Because the cross resistance could affect the efficacy of the insecticides, rotating or mixing insecticides should not use the insecticides with cross resistance. The insecticides affected by different detoxification mechanisms should be incorporated together in resistance management strategies [5]. Besides, Khan et al., found insecticide mixtures could enhance the toxicity of insecticides, such as pyrethroids in the resistant population of house flies [25]. Because the resistance mechanism to each insecticide was independent and initially rare, it would decrease the chances for the occurrence of resistance to both insecticides at the same time [25]. Consequently, insecticide mixtures might be more effective in resistance management programs compared to mosaics or rotational use of these insecticides. Besides, comprehensive preventive and control measures involving chemical, biological, and environmental measures that exerted control over all the life stages of house flies should be used in pest management programs to minimize the selection pressure for resistance. Biological control of organisms was an alternative form of control of the house flies [26, 27], but the resistance of the biorational insecticides should also be noted.

In Zhejiang Province, the resistance to insecticides varied in study districts, depending on which insecticides had been used and how often, how widely, and how long. The most frequently used insecticides showed the greatest ability to induce resistance in the target insects [28]. Totally, the Ningbo strain had the lowest resistance in the past two detections in 2011 and 2014. Besides, the Jiaxing strain was sensitive to three of the five insecticides detected in 2017, except for permethrin with the RR value of 8.1296 and deltamethrin with the RR value of 10.5714, which were relatively low resistance. The insecticides usage in Ningbo and Jiaxing might be more suitable in the management of resistance development. As the Huzhou and Wenzhou strains might have resistance problem to certain insecticides, this should be taken in account in future insecticides application.

As for the criteria, Shah et al. defined RR = 1 as the no resistant population and RR = 2–10 as the very low resistant population [16], and Khan et al. used the criteria that the insect populations with less than 10-fold RRs should be assumed as tolerant rather than resistant [9]; in our study, we defined the resistant population (RR ≥ 5 and the 95% CI did not overlap) according to The test methods of fly resistance to insecticides (GB/T 26350-2010) [15], combining the criterion of Shah et al. [16] to define the high level of resistance. Our standard might be more moderate in defining the resistance population. In our study, we paid more attention to the very high resistance region, because it might be difficult to reverse within a short time and in urgent need of the insecticide adjustment.

In conclusion, our findings confirmed the occurrence of resistance toward permethrin, deltamethrin, beta-cypermethrin, and propoxur in the house flies of Zhejiang Province, while the reversion of the resistance to dichlorvos was found. Continued use of such compounds insecticides would induce resistance in the target insects and influence their control effect. Integration of different insecticides with no cross resistance is a critical approach in integrated pest management strategies. Regular monitoring surveys should be continued to observe the level of the resistance and guide the insecticides usage in pest control. In further study, monitoring and identification of the resistant genes in the house flies will be essential for the management of resistance development.

## Figures and Tables

**Table 1:** LD_50_ and the RR value of the five insecticides in the house flies in Zhejiang Province, China (LD_50_, *μ*g/fly).

City	Year	Dichlorvos			Propoxur			Permethrin			Deltamethrin			Beta-cypermethrin		
		LD_50_	95% CI	RR	LD_50_	95% CI	RR	LD_50_	95% CI	RR	LD_50_	95% CI	RR	LD_50_	95% CI	RR
Susceptible strain	2017	0.7781	0.7018–0.8626	—	0.1550	0.1395–0.1722	—	0.0108	0.0092–0.0127	—	0.0007	0.0006–0.0009	—	0.0090	0.0074–0.0109	—
	2014	0.0453	0.0371–0.0577	—	0.2973	0.2493–0.3543	—	0.0108	0.0092–0.0127	—	0.0009	0.0008–0.0011	—	0.0036	0.0030–0.0043	—
	2011	0.0962	0.0851–0.1076	—	0.0458	0.0396–0.0524	—	0.0124	0.0109–0.0139	—	0.0019	0.0017–0.0023	—	0.0117	0.0103–0.0132	—
Hangzhou	2017	1.0612	0.9162–1.2321	1.3638	8.8094	7.4675–10.3778	56.8348^*∗*^	—	—	—	0.0777	0.0667–0.0904	111.0000^*∗*^	0.1988	0.1725–0.2302	22.0889^*∗*^
	2014	0.3290	0.2832–0.3830	7.2627^*∗*^	>400.0000	—	>1345.4423^*∗*^	—	—	—	0.0696	0.0605–0.0799	77.3333^*∗*^	0.2001	0.1741–0.2308	55.5833^*∗*^
	2011	1.4749	1.2368–1.7588	15.3316^*∗*^	0.2661	0.2303–0.3073	5.8100^*∗*^	—	—	—	0.1045	0.0776–0.1407	55.0000^*∗*^	0.9399	0.7312–1.2081	80.3333^*∗*^
Huzhou	2017	40.4090	29.8130–62.1787	51.9329^*∗*^	23.8774	19.7867–29.2366	154.0477^*∗*^	0.0679	0.0517–0.0889	6.2870^*∗*^	0.1100	0.0781–0.1474	157.1429^*∗*^	0.3224	0.1954–0.4600	35.8222^*∗*^
	2014	0.0053	0.0048–0.0060	0.1170	0.0281	0.0203–0.0409	0.0945	0.0028	0.0010–0.0045	0.2593	0.0021	0.0019–0.0024	2.3333	0.0100	0.0089–0.0011	2.7778
	2011	0.5096	0.4477–0.5802	5.2973^*∗*^	0.1763	0.1384–0.2245	3.8493	0.2001	0.1622–0.2468	16.1371^*∗*^	0.0105	0.0088–0.0127	5.5263^*∗*^	0.0156	0.0127–0.0193	1.3333
Jiaxing	2017	0.4743	0.2437–1.2615	0.6096	0.5654	0.2113–3.3226	3.6477	0.0878	0.0451–0.2263	8.1296^*∗*^	0.0074	0.0045–0.0118	10.5714^*∗*^	0.0146	0.0082–0.0265	1.6222
	2014	0.6746	0.3206–2.1420	14.8918^*∗*^	0.3563	0.1542–1.4583	1.1985	0.0433	0.0252–0.0894	4.0093	0.0052	0.0031–0.0074	5.7778^*∗*^	0.0082	0.0051–0.1450	2.2778
	2011	0.0232	0.1284–0.5067	0.2412	0.0232	0.0158–0.0354	0.5066	—	—	—	0.0105	0.0016–0.1038	5.5263	0.1036	0.0751–1.4900	8.8547^*∗*^
Jinhua	2017	0.5934	0.4955–0.6966	0.7626	0.6838	0.5346–0.7499	4.4116	0.1199	0.0867–0.1294	11.1019^*∗*^	0.0204	0.0179–0.0255	29.1429^*∗*^	0.0691	0.0513–0.0750	7.6778^*∗*^
	2014	0.5491	0.4637–0.6504	12.1214^*∗*^	0.4088	0.3342–0.4913	1.3750	0.0968	0.0684–0.1241	8.9630^*∗*^	0.0182	0.0154–0.0216	20.2222^*∗*^	0.0541	0.0442–0.0659	15.0278^*∗*^
	2011	1.0651	0.9050–1.2401	11.0717^*∗*^	—	—	—	0.1321	0.1082–0.1589	10.6532^*∗*^	0.0220	0.0190–0.0255	11.5789^*∗*^	0.0902	0.0758–0.1068	7.7094^*∗*^
Lishui	2017	—	—	—	>121.5000	—	>783.8710^*∗*^	0.4106	0.3796–0.4462	38.0185^*∗*^	0.0288	0.0129–0.0654	41.1429^*∗*^	0.0030	0.0014–0.0066	0.3333
Ningbo	2014	0.0096	0.0026–0.0490	0.2119	0.0295	0.0115–0.0512	0.0992	0.0048	0.0012–0.0091	0.4444	0.0053	0.0015–0.1270	5.8889^*∗*^	0.0022	0.0003–0.0129	0.6111
	2011	0.0056	0.0013–0.0280	0.0582	0.0115	0.0091–0.0270	0.2511	0.0012	0.0008–0.0010	0.0968	0.0054	0.0016–0.1271	2.8421	0.0022	0.0003–0.0129	0.1880
Quzhou	2017	0.3161	0.2561–0.4002	0.4062	—	—	—	0.3130	0.2525–0.3847	28.9815^*∗*^	0.0491	0.0414–0.0586	70.1429^*∗*^	0.2190	0.1833–0.2706	24.3333^*∗*^
Shaoxing	2017	3.1175	2.8063–3.4663	4.0066	—	—	—	0.1805	0.1548–0.2090	16.7130^*∗*^	0.0646	0.0577–0.0727	92.2857^*∗*^	0.2314	0.1587–0.5060	25.7111^*∗*^
	2014	0.7610	0.6116–0.8881	16.7991^*∗*^	1.0406	0.8586–2.4601	3.5002	0.2430	0.1993–0.2758	22.5000^*∗*^	0.0111	0.0015–0.0231	12.3333^*∗*^	0.1483	0.1038–0.2113	41.1944^*∗*^
	2011	1.1506	1.0305–1.2821	11.9605^*∗*^	—	—	—	0.2079	0.1793–0.2358	16.7661^*∗*^	0.0568	0.0493–0.0644	29.8947^*∗*^	0.4536	0.3958–0.5113	38.7692^*∗*^
Taizhou	2017	0.2583	0.2282–0.2924	0.3320	>110.0000	—	>709.6774^*∗*^	0.1190	0.1042–0.1350	11.0185^*∗*^	0.0602	0.0526–0.0688	86.0000^*∗*^	0.5588	0.4740–0.6525	62.0889^*∗*^
Wenzhou	2017	9.6679	8.1328–11.5459	12.4250^*∗*^	>48.0000	—	>309.6774^*∗*^	0.4253	0.3488–0.5185	39.3796^*∗*^	0.2626	0.2148–0.3187	375.1429^*∗*^	3.2818	2.7617–3.8915	364.6444^*∗*^
	2014	0.1708	0.1146–0.2433	3.7704	1.0634	0.0000–2.5492	3.5769	0.1144	0.0549–0.1672	10.5926^*∗*^	0.0072	0.0011–0.0127	8.0000^*∗*^	0.0214	0.0047–0.0349	5.9444^*∗*^
	2011	0.2616	0.1434–0.4642	2.7193	0.5291	0.2295–0.9901	11.5524^*∗*^	0.1571	0.0849–0.2377	12.6694^*∗*^	0.0098	0.0070–0.0131	5.1579^*∗*^	0.0389	0.0172–0.0672	3.3248
Yiwu	2017	—	—	—	0.7436	0.5956–0.9835	4.7974	0.1313	0.1111–0.1534	12.1574^*∗*^	0.0121	0.0098–0.0145	17.2857^*∗*^	0.0420	0.0376–0.0510	4.6667
	2011	0.9972	0.8861–1.1126	10.3659^*∗*^	—	—		0.1245	0.1038–0.1454	10.0403^*∗*^	0.0374	0.0323–0.0424	19.6842^*∗*^	0.1150	0.0913–0.1357	9.8291^*∗*^
Zhoushan	2017	0.2904	0.2347–0.3647	0.3732	58.8719	37.4590–91.7088	379.8187^*∗*^	0.2395	0.1657–0.3473	22.1759^*∗*^	0.1142	0.0557–0.2041	163.1429^*∗*^	0.2041	0.1340–0.3077	22.6778^*∗*^

^*∗*^Resistant population (RR ≥ 5 and the 95% Confidence interval between the tested strain and the susceptible strain was not overlapped).

**Table 2:** Analysis of the insecticides resistance in different years.

	Dichlorvos	Propoxur	Permethrin	Deltamethrin	Beta-cypermethrin
*χ* ^2^	0.428	7.501	4.528	11.241	0.922
df	2	2	2	2	2
*P*	0.807	0.024^*∗*^	0.104	0.004^*∗*^	0.631

^*∗*^ *P* < 0.05.

**Table 3:** The correlation test of the resistance to different insecticides.

	Dichlorvos		Propoxur		Permethrin		Deltamethrin		Beta-cypermethrin	
	*r*_s_	*P*	*r*_s_	*P*	*r*_s_	*P*	*r*_s_	*P*	*r*_s_	*P*
Dichlorvos	1	—	0.351	0.141	0.271	0.248	0.342	0.102	0.49	0.015^*∗*^
Propoxur	0.351	0.141	1	—	0.711	0.001^*∗*^	0.782	0.000^*∗*^	0.504	0.020^*∗*^
Permethrin	0.271	0.248	0.711	0.001^*∗*^	1	—	0.586	0.004^*∗*^	0.467	0.028^*∗*^
Deltamethrin	0.342	0.102	0.782	0.000^*∗*^	0.586	0.004^*∗*^	1	—	0.734	0.000^*∗*^
Beta-cypermethrin	0.49	0.015^*∗*^	0.504	0.020^*∗*^	0.467	0.028^*∗*^	0.734	0.000^*∗*^	1	—

^*∗*^ *P* < 0.05.

## Data Availability

The data used to support the findings of this study are available from the corresponding author upon request.
